# Medical students’ perceptions about the added educational value of student-run HIV/AIDS educational campaigns in the Dominican Republic

**DOI:** 10.1186/s41256-016-0011-x

**Published:** 2016-08-17

**Authors:** Helena J. Chapman, Jessica Bottentuit-Rocha

**Affiliations:** 1 Department of Environmental and Global Health, College of Public Health and Health Professions, University of Florida, Gainesville, FL USA; 2 School of Medicine, Universidad Nacional Pedro Henríquez Ureña, Santo Domingo, Dominican Republic

**Keywords:** Acquired immunodeficiency syndrome, Dominican Republic, Health education, Medical education

## Abstract

**Objective:**

This purpose of this report was to examine the perceptions of medical students about the strengths, limitations, and recommendations for improvement of the first known student-run HIV/AIDS educational campaigns in the Dominican Republic (DR), as they relate to the added value applied to their educational training.

**Methods:**

A retrospective review was conducted on evaluation reports completed by five medical students who coordinated the implementation of three annual HIV/AIDS educational campaigns in five DR communities, between 2012 and 2014. Thematic analysis was used to identify emerging themes related to perceived strengths, limitations, and recommendations for improvement and develop an acronym related to program strengths as value added to medical education.

**Results:**

Students highlighted that program strengths were the use of social media technology to facilitate communication and culture-based creativity to capture the attention of target audiences; and limitations were inadequate financial support and HIV-related cultural stigma, due to lack of disease knowledge and awareness or perceived contrasts between the federal system and faith-based community. Recommendations for program improvement, such as comprehensive event preparation and knowing the target audience, were described as key to maximizing the delivery of health messages.

**Conclusions:**

Our results highlighted that medical students gained expertise in the effective use of social media technology, culture-based creativity, and team synergy to disseminate HIV/AIDS health information across five DR communities. Students participated in these extracurricular community health campaigns, strengthening skills in communication, health advocacy, and leadership for their medical training. They served as human resources for health and can pave the way as future clinicians and indispensable health educators in local and national health collaborations.

## Introduction

Human immunodeficiency virus (HIV) is one of the “big three” infectious diseases that continues to cause significant morbidity and mortality across the world. In 2015, there were 2.1 million new infections, 36.7 million people living with HIV/AIDS (PLWHA), and 1.1 million deaths from acquired immunodeficiency syndrome (AIDS)-related causes [1]. The island of Hispaniola, comprised by Haiti and the Dominican Republic (DR), remains significantly burdened by the HIV epidemic, with an estimated 72.8 % of HIV cases in the Caribbean (18.0 % in DR, 54.8 % in Haiti) [2]. Although HIV transmission in the DR is more associated with heterosexual intercourse, limited evidence highlights disease burden or risk factors of sexual practices in high-risk groups, including intravenous drug users, men who have sex with men (MSM), commercial sex workers, and batey (sugarcane plantation) residents living in impoverished conditions [3]. In 2012, HIV prevalence estimates varied among adults in the general population (0.8 − 1.0 %) with those of selected vulnerable groups such as MSM (5.2 %), commercial sex workers (4.5 %), and batey residents (2.5 %) [4, 5].

The pandemic requires sustainable political financing and multi-disciplinary health collaborations to reduce cultural, legal, and social barriers and promote equal access to health services, prevention, treatment, and support programs. Agencies, such as United Nations Children’s Fund (UNICEF), Joint United Nations Programme on HIV/AIDS (UNAIDS), Centers for Disease Control and Prevention (CDC), and Presidential AIDS Council (COPRESIDA), provide funding for HIV/AIDS education, research, and social support groups for DR residents. DR health collaborations, however, have not traditionally focused on medical education and training opportunities that aid in the delivery of educational programs and services to local communities. Previous studies that incorporated community health training in medical education have reported that students had increased understanding of health literacy related to nutrition and physical activity [6], influences of social determinants of health on health outcomes [7], and rural community health needs’ assessments [8]. As such, by emphasizing the key approaches of community mobilization and social media strategies in medical education [9], DR medical students can complement their academic learning about preventive medicine in the classroom with community training exercises that integrate medical and public health models.

To meet national health needs and foster the development of national health initiatives by medical students, DR medical students from four universities formed the non-governmental organization, the Dominican Medical Student Organization (ODEM), in 2012. Using the UNAIDS theme, “HIV/AIDS: Getting to Zero”, five medical students coordinated the first known student-run HIV/AIDS educational campaigns for World AIDS Day (WAD) each year between 2012 and 2014. With a team of 65 medical students, they formed activity partnerships with COPRESIDA (Santo Domingo) and Fundación Renacer con Niños Huérfanos del SIDA (FURENIHSI) for children orphaned by AIDS (San Pedro de Macorís). They coordinated educational campaigns in one rural community (Ingenio Santa Fe) in San Pedro de Macorís and four university communities (Gazcue, Herrera, Los Jardines, Zona Universitaria) in Santo Domingo, reaching approximately 7,000 citizens, based on audience attendance at activities and social media promotions. Five communication strategies were used to educate target audiences, including distribution of health pamphlets; informative poster displays; use of social media to disseminate health facts and graphics; organization of health seminars by physicians to medical students and the general community; and community outreach for the administration of gratuitous HIV rapid diagnostic testing with confidential post-test counseling. Topics included HIV/AIDS risk and modes of transmission; knowing HIV status and using safe sexual practices; dispelling myths related to HIV/AIDS; understanding negative effects of stigma or discrimination; and learning about Law 135–11 that promotes human rights, access to healthcare services and treatment for PLWHA [10].

The aim of this report is to examine medical students’ perceptions about the strengths, limitations, and recommendations for improvement of implemented HIV/AIDS educational campaigns, as they relate to the added value applied to their educational training.

## Methods

After the completion of each WAD campaign, each medical student coordinator at participating universities completed a three-section evaluation form: 1) general program information (e.g., date, location, numbers of team and audience members, collaborating persons or agencies); 2) activity description (e.g., objectives, methods, required resources); and 3) activity evaluation (e.g., strengths, limitations, recommendations for improvement). Then, the ODEM national coordinator interviewed each student to confirm details and provide additional feedback for written evaluation reports. These evaluation materials were archived electronically on the ODEM virtual server.

For this study, a retrospective review was conducted on these ODEM evaluation reports, which were completed by five medical students who coordinated the implementation of three WAD campaigns in five DR communities, between 2012 and 2014. Process evaluation strategies [11] were used to assess their perceptions of procedures to program planning and delivery to audiences. Using thematic analysis, both investigators analyzed coded notes for perceived strengths, limitations, and recommendations [12]. They developed themes with illustrating quotations and an acronym to denote program strengths as value added to medical education. This study was reviewed and approved by the Institutional Review Board at the University of Florida (Gainesville, FL, USA) and the Department of Research at the Universidad Nacional Pedro Henríquez Ureña (Santo Domingo, DR).

## Results

The WAD evaluation reports, which were completed by five medical students (1 male, 4 females), described their perceptions about strengths, limitations, and recommendations for improvement related to three WAD campaigns conducted in one rural community (Ingenio Santa Fe) in San Pedro de Macorís and four university communities (Gazcue, Herrera, Los Jardines, Zona Universitaria) in Santo Domingo. Table 1 presents an acronym developed from summarized codes and themes related to these perceived program strengths as value added to medical education.

**Table 1:** Acronym “SYNERGY” that summarizes medical students' skills gained, as perceived by medical student coordinators of HIV/AIDS educational campaigns in the Dominican Republic, between 2012 and 2014

S	Strengthen skills in evidence-based practices for health promotion and disease prevention
Y	Yield dynamic health promotion activities that reflect specific objectives for target audiences
N	Navigate delivery of specific health messages
E	Empower and advocate for health as a human right and equal access to healthcare services
R	Reinforce consistent communication across team members despite geographic distance
G	Generate robust intraprofessional teams with common vision and goals
Y	Yearn to vocalize challenges in event implementation

### Strengths

#### Use of social media technology

Medical student coordinators reported that they gained expertise in developing and disseminating high-quality, low-cost health educational materials and messages to the public by using social media technology like Facebook and Twitter. One student mentioned, “We used the internet to promote our posters, and everyone [used Facebook] to ‘like’ and ‘share’ the information [to the public].” Since they coordinated activities in five communities, the use of social media facilitated team communication and intraprofessional collaborations. Another student stated, “Working with the internet [and social media] allowed us to complete more tasks in less time, where all members could share educational materials prepared by other members.”

#### Creativity matters

Coordinators described that culture-based creativity, or using the imagination to use personal skills and abilities to develop innovative strategies for a specific purpose [13], was fundamental in capturing the attention of target audiences. When organizing professional seminars to university populations, one student recognized that presentations must deliver clear and concise messages: “[The speaker] did not talk about what people already know [about HIV], but rather the actual reality and what is being done.” Another student mentioned the cultural influence in communication skills and the use of colorful clothing that attracts audiences to campaign activities: “Dominicans are like that and when they see something new, [they think] ‘What is that?’ You take advantage and explain [health messages] to them.”

### Limitations

#### HIV-related cultural stigma

Medical student coordinators observed that HIV-related stigma followed a general lack of HIV/AIDS knowledge, including effective treatment, social impact on family, and awareness about personal HIV status. One student commented, “Basically, students enter universities without any knowledge of sexual education”. They described the cultural battle between the federal system and faith-based community, where sexual education programs in primary and secondary schools are limited. Another student stated that condom distribution was not widely accepted as a promotion strategy at universities: “Our medical school did not allow us to distribute condoms inside the institution. So, we moved [our distribution] to the outskirts of the university.”

#### Limited financial support

Coordinators noted that limited financial support was a barrier for funding WAD activities across target communities. This resulted in students using personal funds for educational materials, HIV rapid diagnostic tests, and transportation. With sustainable funding, they believed that they could expand HIV/AIDS educational outreach and screening tests to marginalized communities. One student firmly stated, “We want to keep working hard [on HIV/AIDS outreach] because I believe that it will be something impactful, marking the difference with what we have learned in the medical education model of the Dominican Republic.”

### Recommendations for improvement

#### Foresight in preparation

Medical student coordinators mentioned that comprehensive event preparation is a priority for successful implementation. One student stated, “[I recommend] planning ahead of time and not changing anything at the last moment”. They also identified that marketing of WAD events is crucial to increase audience participation. Another student replied, “People are tired of seeing the same [type of conferences]. We need to develop ‘catchy’ titles and content.”

#### Knowing your target audience

Coordinators described the value in knowing how to captivate and empower audience participation in WAD events. Two students responded, “Many people were curious” and “We observed that audience members left our activities, interested to learn more [about HIV/AIDS].” Since students highlighted positive team synergy as, “With united strength, we can do many great activities,” they suggested that team brainstorming exercises should focus on aligning measurable and achievable project objectives that support specific target audiences.

## Discussion

This is the first known study to explore perceived strengths, limitations, and recommendations of medical students who coordinated the first known student-run HIV/AIDS educational campaigns in the DR. They served as human resources for health in the DR, providing accurate health facts to increase awareness and reduce stigma and erroneous myths about HIV/AIDS while promoting equal access to healthcare services. By using social media technology and culture-based creativity as health promotion tools, they aimed to maximize team synergy and widespread dissemination of WAD events.

By participating in these student-run HIV/AIDS educational campaigns, medical students were able to strengthen the development of the five World Health Organization (WHO)-recognized essential skills of the physician of tomorrow, or the “five-star doctor”: care provider, communicator, community leader, decision-maker, and manager [14]. Ideally, evidence-based preventive health didactic coursework with community training exercises can provide medical students with opportunities to learn and apply reflection learning to their medical education [15]. In contrast with the community health approach as the foundation for all aspects of the Cuban medical curricula [16], only one DR medical school at the time of this study offered first- or second-year community-level rotations in combination with didactic content. Although some U.S. medical schools successfully adapted courses to integrate population health content into basic and clinical science coursework, only three were documented to offer training exercises for skill application within the community or hospital setting during clinical clerkships [17]. Thus, these student-run HIV/AIDS educational campaigns, where no academic credit was granted, demonstrated a stark advantage for DR medical students during their early medical training.

In order to meet the next global health challenges, however, medical schools can use three key strategies to adapt curricula to prepare medical students with community health expertise. First, by focusing on how medical schools should be socially accountable to the local community, curricula can target competencies in holistic health [18], including understanding the influence of social determinants of health on health and well-being. Second, although technological advancements may drive the teaching and learning process to evolve from the traditional classroom model to web-enhanced or blended courses for continued mastery of basic or clinical concepts [19], curricula should still incorporate personalized study, clinical simulations, team-based study, and community-based training for comprehensive learning. Third, the development of health promotion curricula in medical schools will require an evidence-based assessment of established learning objectives [20]. By addressing these approaches, medical schools can provide an enriching academic environment for students to gain lifelong skills in community health, which may directly motivate students to pursue postgraduate training in primary care.

The main limitation of these campaigns was the absence of pre- and post-test evaluation tools to assess effectiveness or overall impact of educational programs based on acquired knowledge of target audiences. Proper health message assessments of interventions are essential to identify best approaches to strengthen programs and empower community members [21]. Thus, future studies may consider a more in-depth formative evaluation for specific needs of each target audience, and subsequent summative evaluation for overall program effectiveness. Also, although evaluation materials were completed by five medical student coordinators, saturation was reached in data analysis, providing a deeper understanding of the educational value related to these three student-run HIV/AIDS educational campaigns.

## Conclusion

During the first known student-run HIV/AIDS educational campaigns, medical students gained expertise in the effective use of social media technology, culture-based creativity, and team synergy to disseminate health information to target audiences in five DR communities. As they developed skills in project coordination and management, they strengthened their leadership experiences in health activism as an added benefit to their medical training. By serving as human resources for health, physicians-in-training can pave the way as future clinicians and indispensable health educators in local and national health collaborations.

## Abbreviations

AIDS, acquired immunodeficiency syndrome; CDC, Centers for Disease Control and Prevention; COPRESIDA, Presidential AIDS Council; DR, Dominican Republic; FURENIHSI, Fundación Renacer con Niños Huérfanos del SIDA; HIV, human immunodeficiency virus; MSM, men who have sex with men; ODEM, Dominican Medical Student Organization; PLWHA, people living with HIV/AIDS; UNAIDS, Joint United Nations Programme on HIV/AIDS; UNICEF, United Nations Children’s Fund; WAD, World AIDS Day; WHO, World Health Organization
